# SARS-CoV-2 spike protein-ACE2 interaction increases carbohydrate sulfotransferases and reduces N-acetylgalactosamine-4-sulfatase by p38 MAPK

**DOI:** 10.1038/s41392-024-01741-3

**Published:** 2024-02-14

**Authors:** Sumit Bhattacharyya, Joanne K. Tobacman

**Affiliations:** Jesse Brown VA Medical Center and University of Illinois at Chicago, Chicago, IL 60612 USA

**Keywords:** Infectious diseases, Biochemistry

## Abstract

Immunostaining in lungs of patients who died with COVID-19 infection showed increased intensity and distribution of chondroitin sulfate and decline in N-acetylgalactostamine-4-sulfatase (Arylsulfatase B; ARSB). To explain these findings, human small airway epithelial cells were exposed to the SARS-CoV-2 spike protein receptor binding domain (SPRBD) and transcriptional mechanisms were investigated. Phospho-p38 MAPK and phospho-SMAD3 increased following exposure to the SPRBD, and their inhibition suppressed the promoter activation of the carbohydrate sulfotransferases CHST15 and CHST11, which contributed to chondroitin sulfate biosynthesis. Decline in ARSB was mediated by phospho-38 MAPK-induced N-terminal Rb phosphorylation and an associated increase in Rb-E2F1 binding and decline in E2F1 binding to the ARSB promoter. The increases in chondroitin sulfotransferases were inhibited when treated with phospho-p38-MAPK inhibitors, SMAD3 (SIS3) inhibitors, as well as antihistamine desloratadine and antibiotic monensin. In the mouse model of carrageenan-induced systemic inflammation, increases in phospho-p38 MAPK and expression of CHST15 and CHST11 and declines in DNA-E2F binding and ARSB expression occurred in the lung, similar to the observed effects in this SPRBD model of COVID-19 infection. Since accumulation of chondroitin sulfates is associated with fibrotic lung conditions and diffuse alveolar damage, increased attention to p38-MAPK inhibition may be beneficial in ameliorating Covid-19 infections.

## Introduction

Immunohistochemistry of post-mortem lung tissue from patients with SARS-CoV-2 infection showed marked decline in intensity and distribution of N-acetylgalactosamine-4- sulfatase (Arylsulfatase B; ARSB) and increase in total chondroitin sulfate by immunohistochemistry.^1^ The mechanisms leading to these observations were not explained by known signaling pathways activated by exposure to coronaviruses. This report addresses the underlying reactions leading to these observations in a cell-based model, using normal, human, primary small airway epithelial cells, treated with the SARS-CoV-2 spike protein receptor binding domain protein and considers the impact of selected therapeutic interventions.

Chondroitin sulfates are vital components of cells and the extracellular matrix (ECM) in all human tissues and throughout living organisms. Chondroitin 4-sulfate (C4S, previously CSA), chondroitin 6-sulfate (C6S, previously CSC), chondroitin sulfate D, and chondroitin sulfate E are linear polymers composed of β-1,4 linked disaccharides made of β-1,3 linked D-glucuronate and N-acetyl-D-galactosamine 4-sulfate (GalNAc4S) residues, with variations in the arrangement of covalently bound sulfate groups. C4S has N-acetyl-D-galactosamine 4-sulfate residues; C6S has N-acetyl-D-galactosamine 6-sulfate residues; chondroitin sulfate D has glucuronate 2-sulfate and N-acetyl-D-galactosamine 4-sulfate residues; and chondroitin sulfate E has N-acetyl-D-galactosamine 4,6-disulfate residues. Dermatan sulfate (DS) consists of disaccharides of iduronate and N-acetyl-D-galactosamine 4-sulfate with β-1-3 linkage; the disaccharides are joined by β-1-4 bonds. Precise determination of the vital roles of chondroitin sulfates in the development and maintenance of cell and tissue structure and function is challenging due to variation in chain length, sulfation sites, proteoglycan partners, and intracellular and extracellular localization. The enzyme N-acetylgalactosamine-4-sulfatase (Arylsulfatase B; ARSB) is required for the degradation of C4S, since ARSB removes the terminal sulfate at the non-reducing end of C4S. ARSB is required for the degradation of C4S and DS, as evident by the accumulation of these sulfated glycosaminoglycans in the congenital disease Mucopolysaccharidosis VI (Maroteaux-Lamy Syndrome), which is caused by innate mutations of ARSB.^2,3^ Accumulation of chondroitin sulfate is recognized as a significant factor in lung fibrosis of different etiologies,^4–7^ and treatment with recombinant ARSB was shown to ameliorate cardiac fibrosis in an animal model.^8^ The regulatory relationship between Retinoblastoma (Rb) and ARSB expression, identified for the first time in this report, provides unexpected insight into the importance of ARSB and its unique modification of chondroitin 4-sulfation.

Chondroitin sulfate biosynthesis requires action by carbohydrate sulfotransferases, such as CHST15 and CHST11. Prior work showed marked increase in expression of the carbohydrate sulfotransferases CHST15 and CHST11 in rat vascular endothelial cells following exposure to Angiotensin (Ang) II and of CHST15 by immunohistochemistry in the vasculature of Covid-19 lung.^1^ Mechanisms for enhanced CHST15 or CHST11 expression or for decline in ARSB following viral infection have not been reported previously, although the role of sulfated glycosaminoglycans, particularly heparin and heparan sulfate, in SARS-CoV-2 viral uptake and in inhibition of viral infection of human cells has been investigated.^9–11^ Biosynthesis of chondroitin sulfate E (CSE) by CHST15 proceeds by transfer of the sulfate residue from 3′-phosphoadenosine-5′-phosphosulfate (PAPS) to the 6-OH of GalNAc4S of C4S.^12^ CHST15 [also known as N-acetylgalactosamine 4-sulfate 6-O-sulfotransferase, B-cell RAG (Recombination Activating Gene)-associated protein, and GALNAc4S-6ST] is required for the synthesis of CSE. Increases in CHST15 or in CSE, have been associated with increased fibrosis in cardiac, lung, and other tissues and with infectivity of dengue virus,^13–18^ as well as in several malignant cells and tissues, including pancreas, ovary, colon, lung, and brain.^19–23^ CHST11 (also known as chondroitin 4-O-sulfotransferase 1 and CHST1) is a carbohydrate sulfotransferase that adds 4-sulfate groups to D-N-acetylgalactosamine residues in C4S or dermatan sulfate.

Many reports have considered how the pathophysiology of Covid-19 relates to interference with the normal balance between effects of Angiotensin (Ang) II and Ang1-7, due to binding of the SARS-CoV-2 spike protein with ACE2 (angiotensin-converting enzyme receptor 2). SARS-CoV-2 spike protein binding with ACE2 can inhibit the ACE2-mediated production of Ang1-7 and lead to imbalance between AngII and Ang1-7/Mas receptor effects. AngII causes vasoconstriction initiated by interaction with the AT1 receptor. Decline in opposing vasodilatation, due to reduced production of Ang1-7, when ACE2 is bound by the SARS-CoV-2 spike protein, may predispose to unopposed signaling events and significant pathophysiology.^24–27^ The impact of inhibition of the renal angiotensin system (RAS) by angiotensin-converting enzyme (ACE) inhibitors or by angiotensin receptor blockers (ARBs) has been considered in relation to treatment and outcome of COVID-19 infection, and studies are ongoing.^28–30^ The experiments presented in this report support a sequence of activation of cellular transcriptional events which proceed from direct effects of the interaction between the spike protein receptor binding domain (SPRBD) and the ACE2 receptor in human airway epithelial cells (AEC). Recognition of the takeover of normal signaling mechanisms by the SARS-CoV-2 spike protein-ACE2 interaction suggests the potential benefit of some targeted pharmacological interventions based on these pathways. Focus on the specific impact of the SPRBD interaction with ACE2 on distinct signaling mechanisms which increase CHST15 and CHST11 expression and inhibit ARSB expression has enabled identification of mechanism-based interventions which may have clinical benefit. Elucidation of the roles of phospho-p38 MAPK, phospho-SMAD3, E2F1, and Rb phosphorylation provides novel insights into how viral usurpation of endogenous signaling pathways can produce sustained pathophysiological consequences in human cells.

## Results

### Increases in chondroitin sulfate and carbohydrate sulfotransferases (CHST15 and CHST11) and decline in N-acetylgalactosamine-4-sulfatase (Arylsulfatase B; ARSB) in airway epithelial cells

**Fig. 1:**
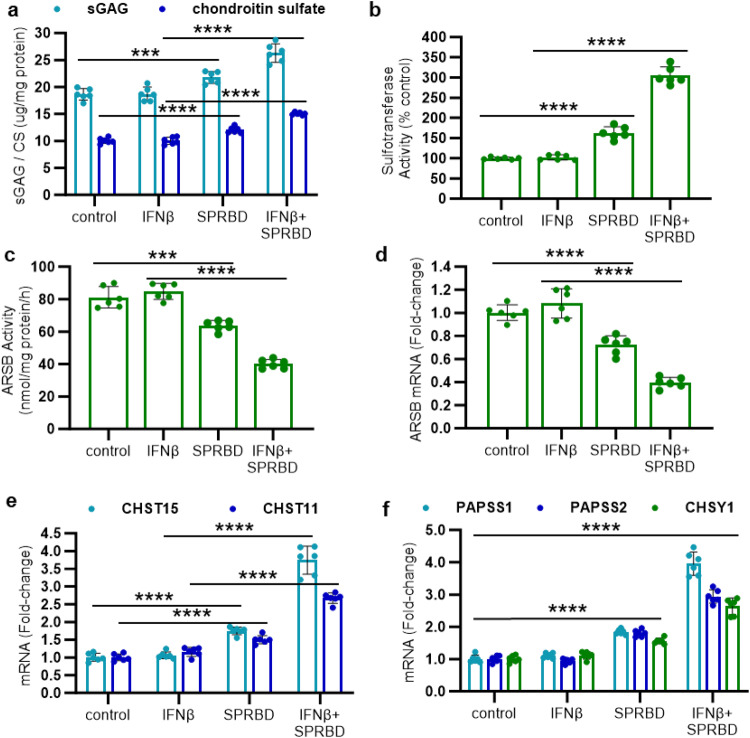
Chondroitin sulfate, total sulfated glycosaminoglycans, Arylsulfatase B, sulfotransferase activity, and expression of chondroitin sulfotransferases and other enzymes for chondroitin biosynthesis. **a** Corresponding to the findings in the infected lung tissue, total chondroitin sulfate (CS) increased by ~2 ug/g protein (*p* = 8.1 × 10^−5^, *n* = 6) and total sulfated glycosaminoglycans increased by over 3 ug/g protein (sGAG) (*p* = 0.0005, *n* = 6) in the AEC following exposure to the SPRBD. Increases are more following combined treatment with SPRBD and IFN-β (>5 µg/g protein for CS, *n* = 6; 7.7 µg/g protein for sGAG, *n* = 6). **b** Consistent with the observed increases in chondroitin sulfate in Covid-19 lung tissue and in the airway cells, sulfotransferase activity increased by 63% following SPRBD and by over 200% following the combination of SPRBD and IFN-β (*n* = 6, *n* = 6). **c** Arylsulfatase B (ARSB) activity declined in the AEC following exposure to the SPRBD (*p* = 0.0006, *n* = 6), and declined over 50% by the combined exposure to SPRBD and IFN-β (*p* = 7.1 × 10^−8^, *n* = 6). **d** The mRNA expression of ARSB also declined (*p* = 6.6 × 10^−5^, *n* = 6) following SPRBD, and declined further following exposure to the combination of SPRBD and IFN-β. **e** Expression of both CHST15 and of CHST11 is significantly upregulated following exposure to the SPRBD and the combination of SPRBD and IFN-β. **f** Expression of the enzymes PAPSS1, PAPSS2, and CHSY1, which are required for chondroitin biosynthesis, increased significantly following exposure to SPRBD (*p* ≤ 2.4 × 10^−6^, *n* = 6) with further increase following IFN-β and SPRBD (*p* ≤ 3.4 × 10^−6^, *n* = 6). All *p*-values were determined using unpaired *t* tests, two-tailed, with unequal variance, and error bars represent one standard deviation. *** represents *p* ≤ 0.001, and **** is for *p* ≤ 0.0001. ARSB arylsulfatase B = N-acetylgalactosamine-4-sulfatase, CHST carbohydrate sulfotransferase, CHSY1 chondroitin sulfate synthase, CS chondroitin sulfate, IFN interferon, PAPSS 3’-phosphoadenosine 5’-phosphosulfate synthase, sGAG sulfated glycosaminoglycan, SPRBD spike protein receptor-binding domain

Normal, primary, human, small airway epithelial cells (AEC) were treated with the SARS-CoV-2 spike protein receptor binding domain (SPRBD; 2.5 µg/ml × 2 h), following exposure to Interferon-β (IFN-β; 10 ng/ml × 24 h). IFN-β was used to increase the expression of angiotensin-converting enzyme 2 (ACE2) receptor, as reported,^31,32^ and thereby amplify the effect of SPRBD and better simulate the impact of viral infection. The expression of ACE2 increased to more than four times the baseline following IFN-β or the combination of IFN-β and SPRBD, and SPRBD alone did not have any significant impact on ACE2 (Supplementary Fig. 1a). Consistent with the previously observed findings from immunohistochemistry,^1^ total chondroitin sulfate (CS) and total sulfated glycosaminoglycans (sGAG) increased significantly following exposure to SPRBD and to the combination of SPRBD and IFN-β (Fig. 1a). CS increased by over 5 µg/mg protein, accounting for over 60% of the increase. Sulfotransferase activity increased by over 200% following exposure to SPRBD and IFN-β (Fig. 1b). ARSB activity (Fig. 1c) and mRNA expression (Fig. 1d) declined following exposure to SPRBD. The mRNA expression of carbohydrate sulfotransferases CHST15 and CHST11 increased to 3.8 and 2.7 times the control values following exposure to SPRBD and IFN-β (*p* < 0.0001, *p* < 0.0001; *n* = 6) (Fig. 1e). The mRNA expression of other enzymes required for chondroitin biosynthesis, including 3’-phosphoadenosine 5’-phosphosulfate synthase (PAPSS1 and PAPSS2), the sulfate donor for sulfotransferase activity, and chondroitin sulfate synthase 1 (CHSY1) also increased significantly following exposure to the SPRBD (Fig. 1f).

### Inhibition of phospho-(Thr180/Tyr182)-p38-MAPK or of phospho-(S423/S425)-SMAD3 blocks increases in CHST15 and CHST11

**Fig. 2:**
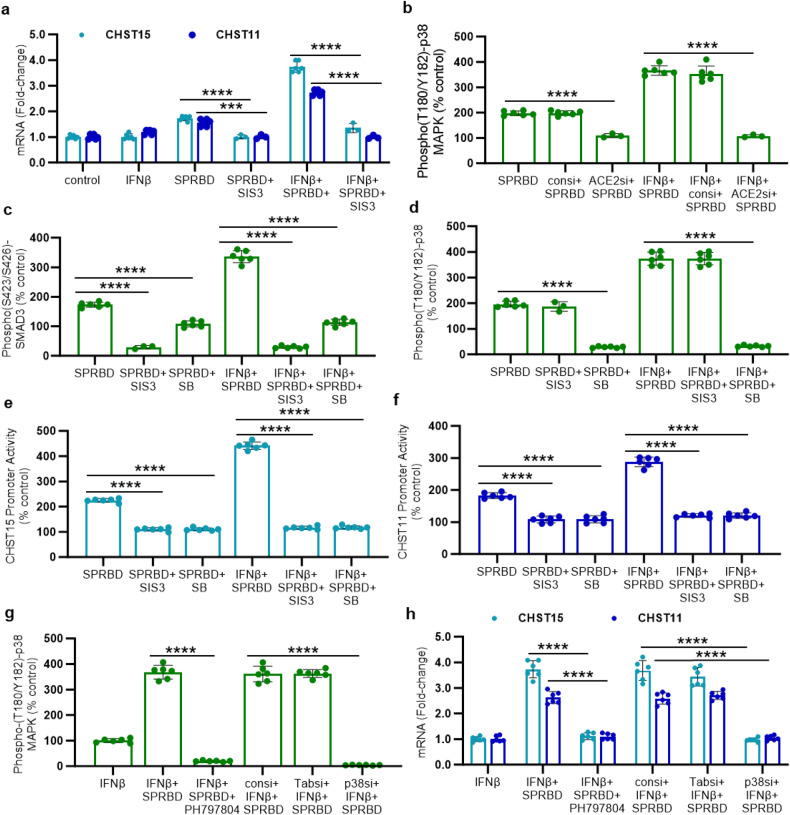
SMAD3 and p38 MAPK inhibitors abrogate the effects of SPRBD. **a** SIS3, a specific inhibitor of phospho-SMAD3, inhibited the SPRBD- and SPRBD + IFN-β- induced increases in expression of CHST15 and CHST11. **b** Following silencing of ACE2 by siRNA, the SPRBD- and SPRBD + IFN-β- induced increases in phospho-(Thr180/Tyr182)-p38 MAPK were inhibited. **c** The SPRBD- and SPRBD + IFN-β- induced increases in phospho-(S423/S425)-SMAD3 were completely inhibited by SIS3 and, to a lesser extent, by SB, the p38 MAPK inhibitor. **d** The SPRBD and SPRBD + IFN-β- induced increases in phospho-(Thr180/Tyr182)-p38 were unaffected by exposure to SIS3 and were inhibited by SB. **e**, **f** Promoter activity of CHST15 and CHST11 was enhanced by exposure to SPRBD and to a greater extent by the combination of IFN-β and SPRBD. Both SIS3 and SB inhibited promoter activation. **g** Treatment of cells with the specific phospho-p38α/β inhibitor PH797804 or with p38α siRNA completely blocked the SPRBD-induced increase in phospho-p38 MAPK detected by ELISA. In contrast, TAB1 siRNA and control siRNA had no effect. **h** The SPRBD-induced increases in expression of CHST15 and of CHST11 were inhibited by p38α siRNA and by PH797804, but not by TAB1 siRNA or control siRNA. All p-values were determined by unpaired *t* test, two-tailed, with unequal variance, and with at least three independent experiments. Error bars represent one standard deviation. *** represents *p* ≤ 0.001; **** is for *p* ≤ 0.0001. ACE angiotensin-converting enzyme, CHST carbohydrate sulfotransferase, NSC NSC23766, SB SB203580, si siRNA, SIS3 specific inhibitor of SMAD3, SMAD Mothers against decapentaplegic homolog 3 (DPC3), SPRBD spike protein receptor-binding domain, TAB1 TGF-beta activated kinase (MAP3K7)

The sequence of treatment of the cultured AEC with SPRBD, IFN-β, enzyme inhibitors, and siRNA is presented in Supplementary Fig. 2a–e and abbreviations are defined in Supplementary Table 1. Effects of inhibitors of cell signaling, including SB203580 (SB), an inhibitor of phospho-(Thr180/Tyr182)-p38 MAPK, and NSC23766 (NSC), a Rho/Rac-1 GTPase inhibitor, were tested, as shown (Supplementary Fig. 3). Marked declines in expression of CHST15 and CHST11 followed exposure to the phospho-(Thr180/Tyr182)-p38 inhibitor, but NSC had no effect. Increases in CHST15 and CHST11 expression were blocked by SIS3, (specific inhibitor of SMAD3) (Fig. 2a). Effectiveness of ACE2 silencing was confirmed by QPCR (Supplementary Fig. 1b). Silencing ACE2 confirmed that ACE2 was required for the observed effect of the SPRBD. ACE2 siRNA blocked the SPRBD-induced increase in phospho-(Thr180/Tyr182)-p38 (Fig. 2b). The increase in phospho-(S423/S425)-SMAD3 following exposure to SPRBD was inhibited by SIS3 (Fig. 2c), and was partially inhibited by SB (Fig. 2c), indicating participation by phospho-p38 in the SPRBD-induced increase in phospho-SMAD3.

Phospho-(Thr180/Tyr182)-p38 MAPK was significantly increased following exposure of the cells to SPRBD (Fig. 2d), and SIS3 had no impact on the increase. Increases in promoter activation of CHST15 (Fig. 2e) and CHST11 (Fig. 2f) were abrogated by treatment with either SIS3 or SB following exposure to SPRBD. Specific inhibition of phospho-p38α by p38α siRNA or by PH797804 blocked the SPRBD-induced increases in phospho-(Thr180/Tyr182)-p38. TAB1 [TGF-beta activated kinase 1 (MAP3K7) binding protein 1] siRNA had no effect (Fig. 2g). SPRBD-induced increases in expression of CHST15 and CHST11 were inhibited by the phospho-p38α inhibitors PH797804 and p38α siRNA, but not by TAB1 siRNA (Fig. 2h).

### Phospho-p38 MAPK mediates decline in ARSB expression through effects on Rb phosphorylation and E2F1

**Fig. 3:**
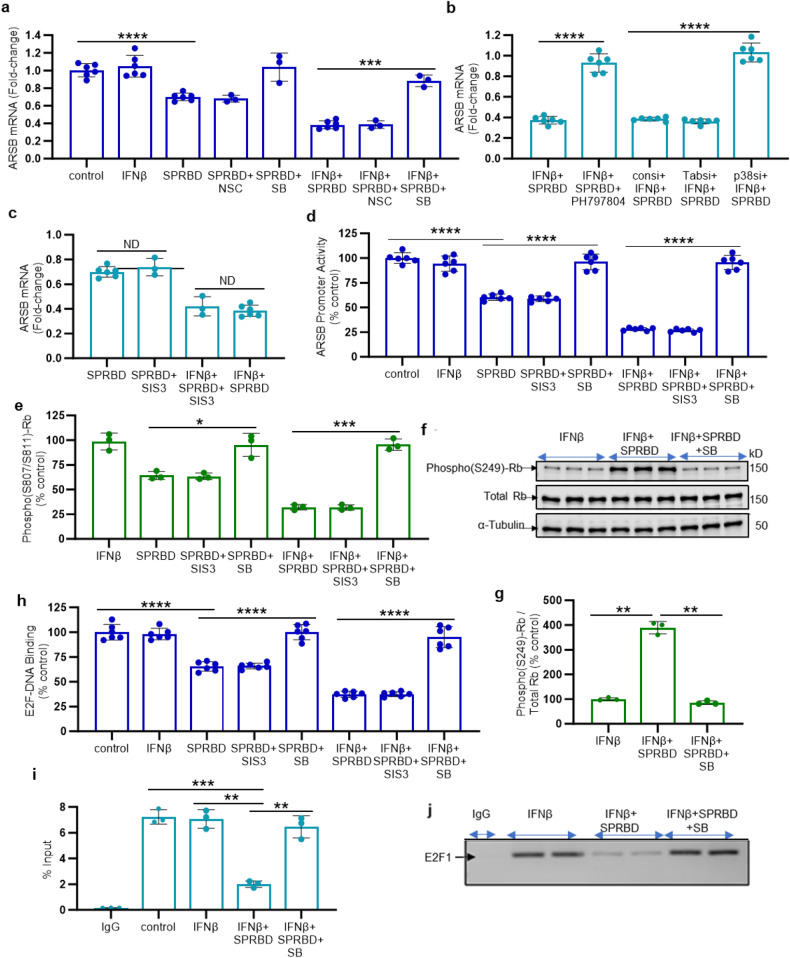
Spike protein receptor binding domain inhibits ARSB activity and expression by activation of phospho-p38 MAPK and phospho-(S249/T252)-RB-E2F1 interaction. **a** ARSB mRNA expression was unaffected by NSC23766, but following exposure to the p38 MAPK inhibitor SB, mRNA expression was restored to baseline control values. **b** In contrast, to the observed effects of p38α inhibitors on expression of CHST11 and CHST15, p38α siRNA and PH797804 reversed the SPRBD-induced decline in ARSB expression. TAB1 siRNA had no effect. **c** SIS3 had no impact on the SPRBD- or SPRBD + IFN-β- induced decline in ARSB expression. **d** ARSB promoter activation was reduced by SPRBD and by SPRBD + IFN-β. These declines were reversed by exposure to SB, but not by SIS3. **e** Following treatment with SPRBD or SPRBD + IFN-β, C-terminal phospho-(Ser807/811)-Rb declined as shown by ELISA. These declines were reversed by SB, but not by SIS3. **f** In contrast to the decline in C-terminal Rb phosphorylation, exposure to SPRBD + IFN-β increased N-terminus phospho-(S249/T252)-Rb, as detected by Western blot and shown in detail in Supplementary Fig. 4a–d. This increase was inhibited by SB, and total Rb was unchanged. **g** Densitometry confirms the impression of Western blot and shows the ratio of phospho-(S249)-Rb to total Rb following SPRBD + IFN-β has increased to 3.89 times the baseline. **h** Following exposure to SPRBD and SPRBD + IFN-β, E2F-DNA binding declined significantly, and SB reversed the declines. **i** %DNA input declined following SPRBD + IFN-β and increased following inhibition of p38-MAPK by SB. **j** Agarose gel of chromatin immunoprecipitation (ChIP) indicated no effect of the IgG negative control on E2F1 binding to the ARSB promoter at baseline, increased binding following IFN-β, reduced binding following SPRBD + IFN-β, and reversal of this decline following treatment with SB. *P* values were determined by unpaired *t* tests, two-tailed with unequal variance, with n of at least 3 independent experiments. Error bars show one standard deviation. * represents *p* ≤ 0.05; ** is for *p* ≤ 0.01, *** for *p* ≤ 0.001, and **** for *p* ≤ 0.0001. ARSB arylsulfatase B = N-acetylgalactosamine-4-sulfatase, IFN interferon, ND no difference, NSC = NSC23766, Rb retinoblastoma protein, SB = SB203580, SIS3 specific inhibitor of Smad3, SPRBD spike protein receptor-binding domain

In contrast to the observed increases in CHST11 and CHST15 (Fig. 1a), mRNA activity and expression of ARSB declined significantly following exposure to SPRBD (Fig. 1c, d), and ARSB expression increased following treatment by SB, the p38-MAPK inhibitor (Fig. 3a). Specific inhibition of p38α-MAPK by siRNA or by PH797804 reversed the SPRBD-induced decline in ARSB expression, but TAB1 siRNA had no effect (Fig. 3b). Neither NSC (Fig. 3a) nor SIS3 (Fig. 3c) reversed the effect of SPRBD on ARSB. ARSB promoter activation was reduced by SPRBD, and treatment with SB reversed the decline (Fig. 3d).

Phospho-p38α was reported to activate N-terminal Retinoblastoma protein (Rb) phosphorylation and increase Rb-E2F1 binding, and to thereby inhibit E2F1-DNA binding.^33,34^ Since the ARSB promoter has several potential E2F1 binding sites,^35^ the potential decline in ARSB expression due to effects of SPRBD-phospho-p38 signaling on Rb N-terminal phosphorylation was addressed. The p38-MAPK inhibitor SB reversed the SPRBD-induced decline in C-terminal phospho-(Ser807/811)-Rb (Fig. 3e). In contrast, SB reduced the SPRBD-induced increase in N-terminal Rb phosphorylation (phospho-Ser249/Thr252), as shown by Western blot (Fig. 3f and Supplementary Fig. 4a–d). The corresponding ratio of phospho-(S249/Thr252)-Rb to total Rb increased following exposure to SPRBD and declined following SB, reflecting the dependence of N-terminal Rb phosphorylation on phospho-p38 (Fig. 3g).

Since N-terminal Rb-phosphorylation, in contrast to C-terminal Rb phosphorylation, is reported to activate Rb binding with E2F1 and reduce availability of E2F for DNA binding, the effect of SPRBD on E2F-DNA binding was assessed. E2F transcription factor-DNA binding assay showed decline following SPRBD-IFN-β exposure, with recovery following SB (Fig. 3h). Chromatin immunoprecipitation (ChIP) assay showed that specific E2F1-binding to the ARSB promoter declined following SPRBD-IFN-β exposure. Percent DNA input declined following SPRBD-IFN-β exposure, and increased following SB (Fig. 3i). Agarose gel indicated that E2F1-DNA binding to the ARSB promoter was reduced by SPRBD-IFN-β exposure and was reversed by SB (Fig. 3j), as shown by densitometry (Supplementary Fig. 5). These findings implicate a complex signaling mechanism whereby phospho-p38 MAPK leads to increased N-terminal Rb phosphorylation, thereby enhancing Rb-E2F1 binding, and negatively regulating the ARSB promoter and suppressing ARSB expression.

### Effects of desloratadine, monensin, and carrageenan on expression of CHST15, CHST11, and ARSB

**Fig. 4:**
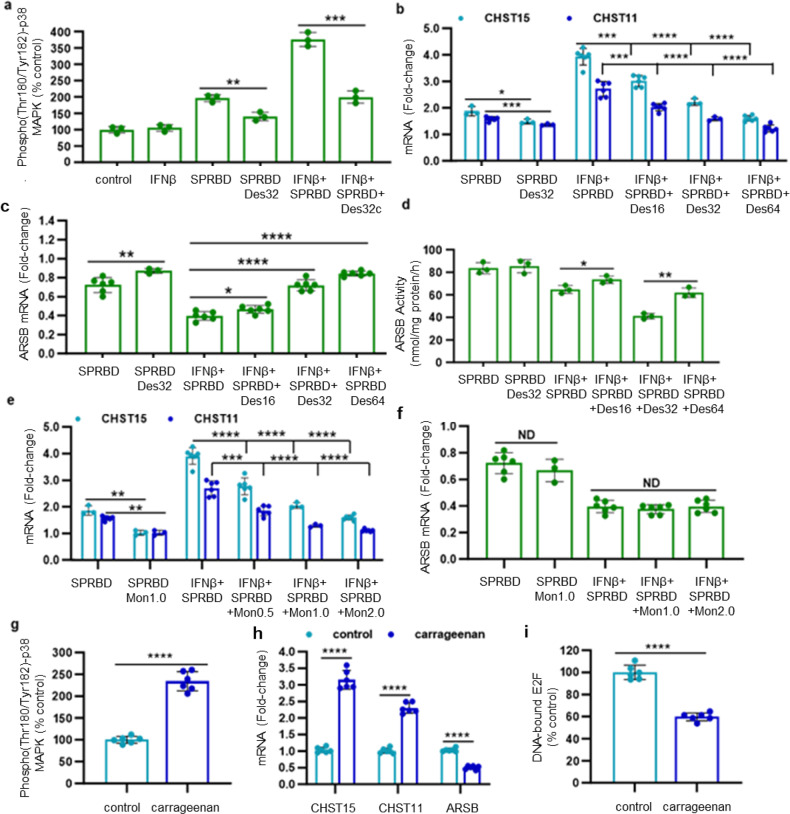
Effects of desloratadine, monensin, and carrageenan on expression of CHST15, CHST11, and ARSB. **a** Exposure to the antihistamine desloratadine at a concentration of 32 µM for 2 h reduced by 62% the SPRBD-induced increase in phospho-(Thr180/Tyr182)-p38 MAPK in the AEC. **b** Consistent with the decline in phospho-p38 MAPK, desloratadine reduced the SPRBD- and SPRBD + IFN-β- induced increases in CHST15 (*p* = 0.04; *p* ≤ 0.0003 with IFN-β) and CHST11 expression (*p* = 0.0008; *p* ≤ 0.0009 with IFN-β, *n* = 6). Declines increased with increasing concentrations of desloratadine from 16 µM to 32 µM to 64 µM, without evidence of cytotoxicity. **c** Fold-change of ARSB mRNA expression increased significantly and progressively with desloratadine 16–64 µM for 2 h (*p* = 0.02, *p* = 1.35 × 10^−6^, *p* = 4.3 × 10^−8^ with IFN-β; *n* = 6). **d** Desloratadine 32 µM for 2 h partially reversed the SPRBD- and SPRBD + IFN-β- induced declines in ARSB activity (from 64.7 to 73.4 nmol/mg protein/h and from 41.0 to 61.8 nmol/mg protein/h with IFN-β; *n* = 3) (*p* = 0.036, *p* = 0.0042, *n* = 3). **e** The polyether antibiotic monensin 1.0 µM for 2 h reduced the SPRBD-induced increases in CHST15 mRNA (*p* = 0.005; *p* ≤ 0.0001 with IFN-β) and CHST11 (*p* = 0.002, *p* ≤ 0.0002 with IFN-β; fold-change compared to control; *n* = 6). **f** Monensin had no significant impact on the SPRBD-induced decline in ARSB. **g** Carrageenan-exposed C57BL/6 J mice had marked increase in phospho-p38 MAPK in lung tissue (*p* = 6.0 × 10^−6^, *n* = 6 per group). **h** mRNA expression of CHST15 and CHST11 was significantly increased in the lung tissue of the carrageenan-exposed mice (*p* < 1 × 10^−5^, *n* = 6). In contrast, ARSB expression declined significantly (*p* = 8.4 × 10^−9^, *n* = 6). **i** Consistent with the phospho-38 MAPK-Rb-E2F1-mediated mechanism of regulation of ARSB mRNA expression, the DNA-bound E2F was significantly reduced (*p* = 1.2 × 10^−6^, *n* = 6) in the lung tissue of the carrageenan-exposed mice. All *p* values were determined using unpaired *t* tests, two-tailed, with unequal variance, and error bars represent one standard deviation. * represents *p* ≤ 0.05; ** is for *p* ≤ 0.01, *** for *p* ≤ 0.001, and **** for *p* ≤ 0.0001. ARSB arylsulfatase B = N-acetylgalactosamine-4-sulfatase, CHST carbohydrate sulfotransferase, Des Desloratadine, IFN interferon, Mon Monensin, ND no difference, PAPSS 3’-phosphoadenosine 5’-phosphosulfate synthase, sGAG sulfated glycosaminoglycan, SPRBD spike protein receptor-binding domain

Therapeutic implications of these chondroitin sulfate-associated signaling pathways were addressed with specific agents known to modulate phospho-p38 MAPK or chondroitin sulfate biosynthesis. Both H1 and H2 antihistamine receptor antagonists have been considered in treatment of Covid-19, including the H1 antihistamine loratadine and its metabolite desloratadine.^36–41^ Treatment of SPRBD-exposed AEC by desloratadine reduced the SPRBD-induced increase of phospho-(Thr180/Tyr182)-p38 MAPK (Fig. 4a) and the mRNA expression of CHST15 and CHST11 (Fig. 4b). Dose effect was apparent; no cytotoxicity was visually observed. Desloratadine also effectively countered the SPRBD-induced declines in ARSB expression (Fig. 4c) and activity (Fig. 4d), showing increased effect at higher doses.

The polyether antibiotic monensin, which has been shown to have effects on sulfated glycosaminoglycan biosynthesis,^42–46^ reduced the SPRBD-induced increases in mRNA expression of CHST15 and CHST11 (Fig. 4e), but had no effect on the SPRBD-induced decline in ARSB (Fig. 4f).

The common food additive carrageenan is well-known for its predictable ability to induce inflammation in animal and cell-based experiments, as documented in thousands of published experiments and in reviews.^47,48^ The impact of algal polysaccharides, including carrageenan, on SARS-CoV-2 infection has been investigated, including in clinical trials.^49–51^ Treatment with the sulfated polysaccharides appears to provide a barrier to impair viral uptake and thereby reduce intracellular viral proliferation.^52^ Interestingly, in 8-week-old C57BL/6J male mice exposed to oral carrageenan in their diet for 60 d, lung tissue showed significant increases in phospho-p38 MAPK (Fig. 4g), and in CHST15 and CHST11 mRNA (Fig. 4h). In contrast, expression of ARSB declined (Fig. 4h). Consistent with the proposed p38 MAPK-mediated mechanism presented in Fig. 3, the DNA-bound E2F was significantly reduced (Fig. 4i). These findings suggest that both SARS-CoV-2 and carrageenan induce disruption of vital cell signaling transduction pathways which may be amenable to treatment by interruption of p38 MAPK signaling.

## Discussion

**Fig. 5:**
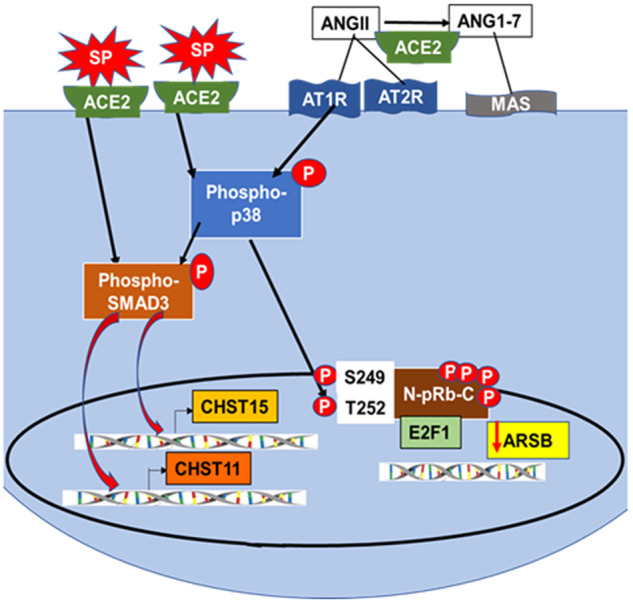
SARS-CoV-2 spike protein binding with ACE2 initiates transcriptional events which modify chondroitin sulfation through activation of phospho-p38 MAPK. This schematic presents the signaling mechanism by which the SARS-CoV-2 spike protein receptor-binding domain (SPRBD) interacts and initiates the transcriptional events which lead to increased chondroitin sulfate in airway epithelial cells and in Covid-19 infected lung.^1^ The activation of phospho-p38 MAPK and phospho-SMAD3 lead to increased expression of CHST15 and CHST11. Phospho-p38 MAPK leads to N-terminal phosphorylation of Rb and enhanced Rb binding with E2F1, which reduces ARSB promoter binding with E2F1 and reduces ARSB expression. Additional effects may arise from ACE2-SPRBD binding, due to reduced availability of ACE2 for other biochemical reactions, including the conversion of AngII to Ang1-7. AngII interaction with the angiotensin II receptor type 1 (AT1R) increases phospho-p38 MAPK, and increased availability of AngII may contribute to the observed downstream effects of p38 MAPK on transcription. The altered balance between AngII-induced vasoconstriction and Ang1-7 vasodilatation, following interaction with the G-protein coupled Mas receptor, may have additional pathophysiological consequences. ACE2 angiotensin-converting enzyme 2, AngII angiotensin II, ARSB arylsulfatase B, AT1R angiotensin II receptor type 1, AT2R angiotensin II receptor type 2, CHST carbohydrate sulfotransferase, Mas Ang1-7 receptor, pRb retinoblastoma protein, SMAD Suppressor of Mothers against Decapentaplegic, SPRBD SARS-CoV-2 spike protein receptor binding domain

The in vitro effects following exposure of cultured, normal, human, small airway epithelial cells (AEC) to the SARS-CoV-2 spike protein binding domain (SPRBD) indicate increased expression of carbohydrate sulfotransferases CHST15 and CHST11 and other chondroitin sulfate biosynthetic enzymes, increased sulfotransferase activity, increased content of chondroitin sulfate, and declines in ARSB expression and activity. Reduced expression of ARSB leads to the accumulation of chondroitin sulfate, since hydrolysis of the 4-sulfate group is required for chondroitin 4-sulfate degradation.^53–56^ Both the increases in CHST15 and CHST11 and the decline in ARSB are mediated by phospho-p38 MAPK. SMAD3 is required for CHST15 and CHST11 expression, whereas the expression of ARSB is regulated by N-terminus Rb phosphorylation-E2F1 interaction (Fig. 5). Previously reported studies of N-terminal Rb phosphorylation presented a complex mechanism by which Rb-E2F binding increased, rather than decreased as occurs by C-terminal Rb-phosphorylation.^33,34^ Application of this mechanism of Rb activation by N-terminal phosphorylation, which overrides Rb inhibition by C-terminal phosphorylations, provides a novel, coherent approach to how ARSB expression may be down-regulated following stimulation of phospho-p38 by SPRBD-ACE2 in the AEC.

Study results indicate that pathways leading to decline in ARSB and increases in expression of CHST15 and CHST11 following exposure to the SPRBD require activation of phospho-p38 MAPK. Phospho p38-MAPK has been implicated in critical interactions among exogenous exposures, intracellular signaling, and transcriptional events in a wide range of experiments.^57–60^ TAB1 has been reported to participate in the activation of phospho-p38α following exogenous stimulation,^61–63^ but silencing TAB1 did not affect phospho-p38 activation in the experiments in this report (Figs. 2g, h, 3b). Crosstalk between the p38 and Smad pathways has been reported in fibrogenic signaling following AngII and downstream of TGF-β,^64–66^ and the experiments in this report support their interaction. The current experiments provide novel insight into how both SMAD3 and phospho-p38 MAPK participate in the regulation of the biosynthesis and maintenance of 4-sulfated chondroitins. Other work has shown that when ARSB is inhibited and 4-sulfation sustained, SHP2 binding to chondroitin 4-sulfate is increased, leading to reduced SHP2 activity and sustained phosphorylation of ERK1,2, JNK, and p38.^3,67–70^ The impact of phospho-38-MAPK on N-terminal Rb phosphorylation, Rb-E2F1 binding, and suppression of ARSB promoter activation, may be part of a loop, in which p38 phosphorylation is sustained, due to enhanced chondroitin 4-sulfate - SHP2 binding which follows decline in ARSB and increased chondroitin 4-sulfation.

Increased understanding of how chondroitin sulfate is involved in the pathogenesis of Covid-19 may help in the development of preventive and therapeutic strategies. The potential benefit of the H1 histamine receptor blocker Desloratadine is suggested by the measured decline in phospho-p38 and by inhibition of the SPRBD effects on expression of CHST15, CHST11 and ARSB, as well as by pharmacological studies that identified desloratadine/loratadine as a mechanism-based target.^36–41^ Several clinical studies of H2 blockers on the response in Covid- 19 have been reported and additional studies of antihistamines are ongoing.^71–73^

Analysis of intensity and distribution of total chondroitin sulfate, CHST15 and ARSB immunohistochemistry in sections from SARS-CoV-2-infected lungs demonstrated marked increase in chondroitin sulfate and prominence of vascular-associated CHST15, in contrast to decline in ARSB.^1^ These findings were similar to those observed in diffuse alveolar damage from other causes and suggest that accumulation of chondroitin sulfate might be a significant component in refractory lung disease and pulmonary fibrosis. Previously, decline in ARSB was implicated in the response to hypoxia in human bronchial epithelial cells^74^ and in failure of patients with moderate COPD to respond to oxygen therapy.^75^ These findings provide additional evidence that decline in ARSB contributes to refractory clinical response in SARS-CoV- 2 infection. By use of the original SPRBD sequence which binds with ACE2, the experiments in this report have focused on how disruption of normal ACE2 function by the SARS-CoV-2 - ACE2 interaction affects signaling in normal, human, airway cells. In this model, viral uptake does not occur, and other spike protein mediated interactions, such as with TMPRSS2, are not anticipated. The original WT sequence differs from subsequent variants, such as the Omicron variant, by about 15 substitutions within the SPRBD.^76,77^ These substitutions are reported to enhance binding with ACE2 and reduce viral uptake and disease severity.^78–80^

The precise mechanism by which the SPRBD-ACE2 interaction leads to activation of phospho-p38-MAPK is not yet clarified. Interception of the activation of p38-MAPK emerges as a therapeutic goal, and H1-receptor blockers may directly inhibit phospho-p38 activation and, thereby, prevent the effects of increased expression of CHST15 and CHST11, of the reduced expression of ARSB, and of the accumulation of chondroitin sulfate following SARS-CoV-2 infection. Also, atypical antibiotics, such an monensin, which directly affect chondroitin 4-sulfate and dermatan sulfate biosynthesis, may provide new targets to disrupt virus-initiated signaling. Increased attention to the impact of chondroitin sulfates and ARSB in Covid-19 pathobiology and to the role of phospho-p38 activation in the mechanisms of their expression may yield new tools and new insights which will reduce the morbidity and mortality of Covid-19.

## Materials and methods

### Cell culture and cell treatments

Normal, primary, human, small airway epithelial cells (PCS-301-010, ATCC, Manassas, VA, USA) were grown in Airway Epithelial Cell Basal Medium (PCS-300-030, ATCC) supplemented with Bronchial Epithelial Cell Growth Kit (PCS-300-040, ATCC) and Penicillin-Streptomycin (penicillin 10 U/ml; streptomycin 10 µg/ml, ATCC), per recommendations. The cells were initially grown in T25 flasks (Falcon 353109, Thermofisher, Waltham, MA, USA) and sub-cultured in 24-well plates (Falcon 353047, Thermofisher) for experiments. Cells were maintained at 37 °C in a humidified 5% CO_2_ environment, and media were changed every two days. The treatments were diluted in basic media without any additive and added directly to the cultured cells. Cell preparations were treated with Interferon-β (IFN-β; 10 ng/ml; Sigma-Millipore, St. Louis, MO, USA), a known stimulator of angiotensin-converting enzyme 2 (ACE2) expression^31,32^ (Supplementary Fig. 1a). Following exposure to IFN-β for 22 h, some cell preparations were then treated with inhibitors for four hours. Inhibitors included: p38 MAPK Inhibitor SB203580 (SB; Adezmapimod, Tocris Bioscience, Bio-Techne, St. Minneapolis, MN, USA; 10 µM); p38α/p38β inhibitor PH797804 (Selleck Chemicals, Houston, TX, USA; 10 µM); Specific Inhibitor of SMAD3 (SIS3, Tocris, Bio Techne Corporation, Minneapolis, MN, USA; 3 µM); Rho/Rac1 inhibitor NSC23766 (NSC; Tocris; 1 ng/ml); Desloratadine (Sigma-Millipore, St. Louis, MO, USA; 16 µM, 32 µM, 64 µM), a histamine H1 receptor antagonist, reported to activate p38-MAPK;^36^ and Monensin (Sigma-Millipore; 0.5 µM, 1 µM, 2 µM), a polyether antibiotic which inhibits synthesis of dermatan sulfate and chondroitin sulfate.^42–46^ The cells were then treated for two hours with purified recombinant SARS-CoV-2 Spike Protein-Receptor Binding Domain protein (SPRBD; AA: Lys310-Leu560; QHD43416; Amsbio, Cambridge, MA, USA; TP750182, 2.5 µg/ml), which was expressed in *E.coli* cells. The sequence is presented in Supplementary Fig. 6. Cell preparations were treated with inhibitors for four hours in combination with IFN-β and for two hours in combination with SPRBD. The schedules for cell treatments with siRNA, IFN-β, SPRBD, and enzyme inhibitors and for media exchange are shown in Supplementary Fig. 2a–e. Some cell preparations were treated only with inhibitors and SPRBD without IFN-β. Other cell preparations were treated with siRNA for 24 h prior to other exposures, then media were exchanged, and cells were treated with IFN-β or directly with inhibitors, followed by SPRBD exposure. Cells were harvested at 26 h, or at 50 h, if treated with siRNA.

### Animal care and carrageenan exposure

Eight-week-old male C57BL/6J mice (*n* = 12) were purchased (Jackson Laboratories, Bar Harbor, Maine, USA) and housed in the Veterinary Medicine Unit at the Jesse Brown VA Medical Center (Chicago, IL, USA). All procedures were approved by the Animal Care Committees of the University of Illinois at Chicago and the JBVAMC in accord with the Institutional Animal Care and Use Committee (IACUC) standards. Mice were fed a standard diet and maintained in individual cages with routine light-dark cycles.^81^ After acclimatizing to the environment, the water supply was changed to ddH_2_O with undegraded carrageenan (λ–κ carrageenan 10 mg/l; Sigma Chemical Co., St Louis, MO, USA; n = 6) or without carrageenan (*n* = 6). Weight and water consumption were measured weekly; animals ate standard mouse chow (65% carbohydrate, 20% protein and 15% fat) and drank water ad libitum. No effects of carrageenan on body weight, activity, or well-being were observed. Mice were euthanized on d60 and lung and other organs were harvested and frozen at −80 °C.

### ELISAs for phospho-Thr180/Tyr182-p38 MAPK, phospho-(S423/S425)-SMAD3 ELISA, and phospho-(Ser807/S811)-Rb

Cell extracts were prepared from both treated and untreated control airway epithelial cells (AEC) in cell lysis buffer (Cell Signaling Technology, Danvers, MA, USA; 9803S). Phospho-p38 MAPK was measured in cell samples using a Pathscan® Phospho-p38 MAPK Thr180/Tyr182 Sandwich ELISA (Cell Signaling). Samples were added to the wells of a microtiter plate precoated with a capture antibody to phospho-(Thr180/Tyr182)-p38 MAPK. The captured phospho-(Thr180/Tyr182)-p38 MAPK was detected by a p38 MAPK detection antibody. A horseradish peroxidase (HRP)-linked anti-rabbit antibody was used to recognize the bound detection antibody, and hydrogen peroxide/tetramethylbenzidine (TMB) substrate was used to develop color proportional to the bound HRP. The reaction was stopped, and the optical density of the color was read at 450 nm in a plate reader (FLUOstar, BMG, Cary, NC, USA). The optical densities were normalized by total protein of the cell extract and expressed as percentage of control.

Phosphorylation of SMAD3 in the treated or control nuclear extracts was determined by using a phospho-(S423/S425)-SMAD3 SimpleStep ELISA (Abcam, Waltham, MA, USA), which employs a labeled capture and detector antibody to measure the phospho-SMAD3 in the sample. Nuclear extracts were prepared using a nuclear extraction kit (Active Motif, Carlsbad, CA, USA; 40010). The entire complex was immobilized in the plate wells by the anti-tag antibody. The bound HRP activity was measured by developing color with hydrogen peroxide/TMB substrate, and the absorbance was measured at 450 nm in a plate reader. The sample values were extrapolated from the standard curve and normalized with total protein in the nuclear extracts.

Pathscan® ELISA for phospho-(Ser807/S811)-Rb protein was procured (Cell Signaling), and C-terminal phosphorylation of Rb protein was determined in the cell extracts of treated and control AEC according to the protocol. Phospho-(Ser807/811)-Rb protein in the samples was captured by a precoated capture antibody in the wells of the microtiter plate. The captured phospho-(Ser807/811)-Rb protein was further detected by a detection antibody and bound detection antibodies were identified by an HRP-linked secondary antibodies. HRP activity was quantified using hydrogen peroxide/TMB substrate. The enzymatic reaction was stopped by acidic stop solution, and color was measured at 450 nm in a plate reader (FLUOstar). The optical densities were normalized by the total protein of the cell extract and expressed as percentage of control.

### CHST15, CHST11 and ARSB promoter activity by Luciferase reporter assay

Promoter activity following treatment of the AEC with SPRBD was determined by human CHST15, CHST11 and ARSB promoter constructs in a *Renilla reniformis* luciferase reporter gene (Ren SP; OriGene, Rockville, MD, USA). Transfections were performed with cells at 70% confluence, using FuGENE HD transfection reagent (Promega, Madison, WI, USA). The β-actin promoter (GoClone) construct (SwitchGear Genomics, Active Motif, Carlsbad, CA, USA) was used as a positive control, and a scrambled sequence (R01) with the *Renilla* luciferase reporter was a negative control. After 24 h incubation, luminescence was read in a microplate reader (BMG). The changes in luminescence due to modifications in binding to the CHST15, CHST11 or ARSB promoter constructs were detected by the LightSwitch assay system (SwitchGear Genomics).

### N-acetylgalactosamine-4-sulfatase (Arylsulfatase B; ARSB) activity assay

ARSB activity measurements were performed using a fluorometric assay, following a standard protocol with 20 μl of cell homogenate prepared in ddH_2_O on ice and 80 μl of assay buffer (Na acetate 50 mM with barium acetate 20 mM, pH 5.6) with 100 μl of the exogenous substrate [5 mM 4-methylumbelliferyl sulfate (MUS) in assay buffer] in a black microplate.^3^

### Measurement of total sulfated glycosaminoglycans (sGAG) and chondroitin sulfate

Total sulfated glycosaminoglycans (sGAG) were measured in the cell extracts by sulfated GAG assay (Blyscan^™^, Biocolor Ltd, Newtownabbey, Northern Ireland).^82^ This assay uses 1,9-dimethylmethylene blue to detect chondroitin 4-sulfations, chondroitin 6-sulfations, keratan sulfate, heparan sulfate, and heparin, but not hyaluronan or disaccharides. Total chondroitin sulfate (CS) in the samples was determined following immunoprecipitation with a chondroitin sulfate antibody (CS-56, Abcam, Waltham, MA, USA). Dynabeads (Life Technologies, Carlsbad, CA, USA) were coated with CS-56 antibody, and beads were mixed with samples, incubated, and immunoprecipitated, as previously. Immunoprecipitated CS molecules were eluted and subjected to the Blyscan sulfated GAG assay, as above.

### Silencing of ACE2, p38α-MAPK, and TAB1 expression by siRNA

Small interfering (si) RNAs were used to silence human ACE2 (NM_021804; Qiagen, Germantown, MD, USA), TAB1 (MAP3K7IP1; NM_006116, NM_153497; Invitrogen, Waltham, MA, USA) and p38α (MAPK14; NM_001315, NM_139012, NM_139013, NM_139014; Invitrogen), and control siRNA (Qiagen). Some cell preparations were treated for 24 h, prior to the addition of test reagents. Effectiveness was determined by quantitative PCR. Cells were grown to ~70% confluency and silenced by adding 0.6 μl of 20 μM siRNA (150 ng), mixed with 100 μl of serum- free medium and 12 μl of HiPerfect Transfection Reagent (Qiagen).

### Total sulfotransferase activity

Total sulfotransferase activity was determined using the Universal Sulfotransferase Activity kit (EA003; Bio-Techne Corp., Minneapolis, MN, USA) which detects sulfotransferase activity and uses 3ʹ-phosphoadenosine-5ʹ-phosphosulfate (PAPS) as the donor substrate. Inositol monophosphatase 3 (IMPAD1) is used as a coupling phosphatase to remove inorganic phosphate quantitatively from the nucleotide 3ʹ-phosphoadenosine-5ʹ-phosphate (PAP) which is generated during the sulfotransferase reaction when PAPS converts to PAP. The coupling 3’-phosphatase releases one inorganic phosphate from PAP which is detected using malachite green reagents. Activity is normalized using total cellular protein and expressed as percentage of control.

### mRNA expression of CHST15, CHST11, ARSB, ACE2, PAPSS1, PAPSS2, CHSY1

Total RNA was prepared from treated and control cells using an RNeasy Mini Kit (Qiagen). Equal amounts of purified RNAs from the control and treated cells were reverse-transcribed and amplified using Brilliant SYBR Green QRT-PCR Master Mix (Bio-Rad, Hercules, CA). Human β-actin was used as an internal control. QRT-PCR was performed using the following specific primers:

CHST11 human (NM_018413) forward: 5ʹ-GTTGGCAGAAGAAGCAGAGG-3ʹ and reverse: 5ʹ- GACATAGAGGAGGGCAAGGA-3ʹ;

CHST15 human (NM_015892) forward: 5ʹ-ACTGAAGGGAACGAAAACTGG-3ʹ and reverse: 5ʹ- CCGTAATGGAAAGGTGATGAG-3ʹ;

ARSB (NM_000046)forward: 5ʹ-AGACTTTGGCAGGGGGTAAT-3ʹ and reverse: 5ʹ- CAGCCAGTCAGAGATGTGGA-3ʹ;

ACE2 (NM_001371415, NM_021804) forward: 5ʹ-CGAAGCCGAAGACCTGTTCTA-3ʹ and reverse: 5ʹ-GGGCAAGTGTGGACTGTTCC-3ʹ;

CHSY1 (NM_014918) forward: 5ʹ-CGA CAG GAA CTT TCT CTT CGT GG-3ʹ and reverse: 5ʹ-CGT ACA GAT GTG TCA GAA CCC TC-3ʹ;

PAPSS1 (NM_005443.5) forward: 5ʹ-GAC GAT GTT CCT TTG ATG TGG-3ʹ and reverse: 5ʹ-RIGHT TCC TTC CCT GTT TCT GGA TG-3ʹ;

PAPSS2 (NM_004670.4) forward: 5ʹ-AGA AGC AAA AGA CGG AGA ACC-3ʹ and reverse: 5ʹ-TTC CAG CAC CAG AGA GAC CT-3ʹ.

Cycle threshold (Ct) was determined during the exponential phase of amplification and relative expression was expressed using β-actin control.^83^ Primers were designed using Primer 3.^84^

### Western blot of phospho-(Ser249/Thr252)-Rb protein

Cell lysates were prepared from control and treated cells in cell lysis buffer (Cell Signaling) with protease and phosphatase inhibitors (Halt Protease and Phosphatase Inhibitor Cocktail, Thermo Scientific, Pittsburgh, PA, USA). Western blots were performed on 10% SDS gels with commercial antibodies to phospho-(Ser249)-Rb protein (Abcam), Rb Protein (Santa Cruz Biotechnology, Santa Cruz, CA, USA), and α-tubulin (Abcam) to probe for the proteins of interest by established procedures. Immunoreactive bands were visualized using enhanced chemiluminescence (Clarity Western ECL Substrate; Bio-Rad, Hercules, CA, USA) in Odyssey ® XF Imaging System (LI-COR Biosciences, Lincoln, NE, USA). The optical density of immunoreactive bands was measured, and the optical densities of the phospho-(Ser249)-Rb protein bands were normalized with the optical densities of Rb protein and α-tubulin. The density of treated and control samples was then compared.

### E2F transcription factor (TF) filter plate assay

TF Filter Plate Assay kit and specific DNA probe for E2F were obtained (Signosis, Santa Clara, CA, USA). Nuclear extracts from treated and control cells were prepared using a nuclear extraction kit (Active Motif; 40010). They were mixed with a specific biotin-labeled E2F DNA binding sequence and formed TF-DNA complexes. A filter plate was used to retain the bound DNA probe and remove free probe. The bound pre-labeled DNA probe was then eluted from the filter and collected for quantitative analysis through DNA plate hybridization. The captured DNA probe was further detected with streptavidin-HRP. Luminescence was reported as relative light units (RLUs) by a microplate reader, and the luminescence was normalized by the total protein of the nuclear extract and expressed as percentage of control.

### Chromatin immunoprecipitation (ChIP) of E2F1 and ARSB promoter

ChIP assay was performed utilizing a ChIP assay kit (ChIP IT Express Kit; Active Motif; 53008). Control and treated AEC cells were fixed with 1% formaldehyde which was diluted in cell basal media without additives for 10 min at room temperature, followed by shearing of chromatin by sonication. Sheared DNA was incubated with anti-E2F antibodies (Cell Signaling) for 1 h. Protein–DNA complexes were precipitated by protein A/G-coupled agarose beads. After purification of the DNA from the immunoprecipitated complexes by reversal of cross-linking, followed by proteinase K treatment, real-time RT-PCR was performed using SYBR Green QRT-PCR Master Mix (Bio-Rad). QRT-PCR was performed using the following specific ARSB promoter primers: Left Primer: 5’-TTAGACCTCTGCCTTTTCACC-3’; Right Primer: 5’-CCTGTAAAGCCTGCAACAAC-3’. These primers encompassed the putative E2F1 binding element in the ARSB promoter (SwitchGear Genomics). Band intensity was compared between the treated and the control samples on a 2% agarose gel.

### Statistical analysis

Data presented are the mean ± SD of at least three independent experiments. Statistical significance was determined by unpaired *t* tests, two-tailed, and corrected for unequal standard deviations using Microsoft Excel, InStat3, or Prism 9.5.1 software (GraphPad, La Jolla, CA, USA). In the figures, error bars show one standard deviation, and the bar height represents the mean value. Individual dots represent unique experimental determinations. Statistical significance is presented with exact *p* values or asterisks, with * for *p* < 0.05, ** for *p* ≤ 0.01, *** for *p* ≤ 0.001, and **** for *p* ≤ 0.0001.

### Supplementary information

Supplementary Figures and Table

## Data Availability

Data are available by communication with JKT.
