# Supplementary material for: SARS-CoV-2 spike protein-ACE2 interaction increases carbohydrate sulfotransferases and reduces N-acetylgalactosamine-4-sulfatase by p38 MAPK
Source: Signal Transduct Target Ther. 2024 Feb 14;9:39. doi: 10.1038/s41392-024-01741-3 (PMC10866996; doi:10.1038/s41392-024-01741-3)
Supplement: Supplementary file 1 — Supplementary Figures and Table [file 41392_2024_1741_MOESM1_ESM.docx]

Supplementary Materials for

# SARS-CoV-2 spike protein-ACE2 interaction increases carbohydrate sulfotransferases and reduces N-acetylgalactosamine-4-sulfatase by p38 MAPK

Sumit Bhattacharyya, Ph.D. and Joanne K. Tobacman, M.D.

Correspondence to: jkt@uic.edu

**This PDF file includes:**

Figures S1, S2, S3, S4, S5, S6

Table S1

**Fig.S1 Effects of IFN-β and ACE2 siRNA.**


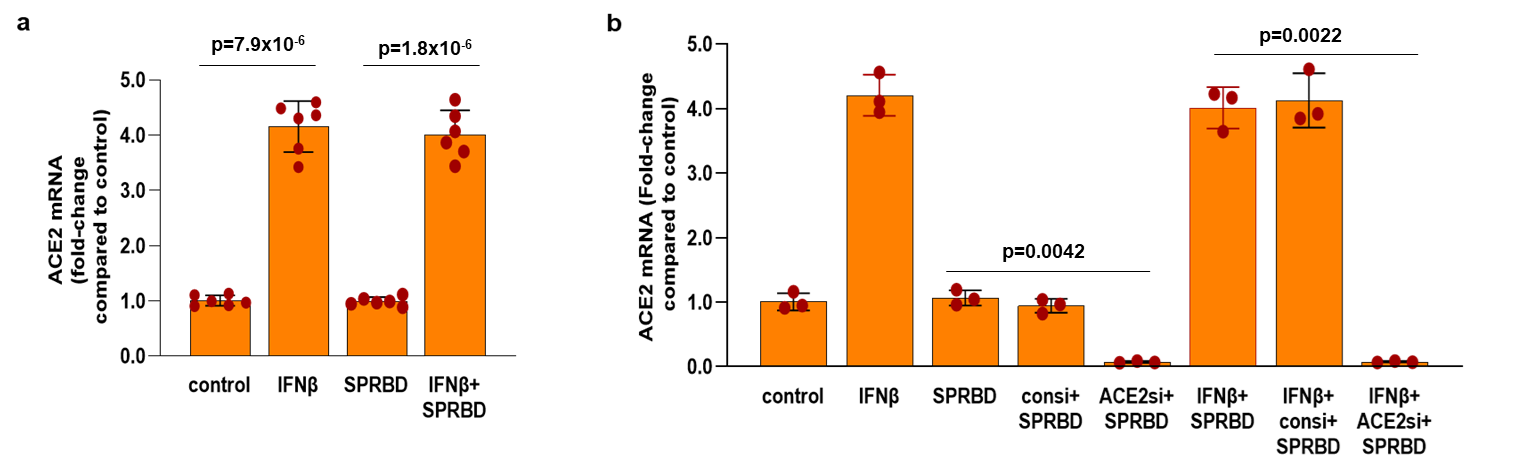


**a.** Treatment of the cultured AEC with Interferon(IFN)-β amplified the impact of SPRBD by increasing the expression of ACE2 by more than 4 times the baseline level (p<1x10^-4,^ n=6).

**b.** Expression of ACE2 was inhibited by ACE2 specific siRNA following exposure to SPRBD with or without IFN-β.

[ACE2=angiotensin converting enzyme 2 receptor; ACE2si=ACE2 siRNA; AEC=airway epithelial cells; consi=control siRNA; IFN=interferon; Rx=therapy; SPRBD=spike protein receptor binding domain]

Fig.S2 Schedule of exposure to siRNA, IFN-β, SPRBD, enzyme inhibitors, and treatments

**Fig.S2a-S2e. Schematic showing the sequence and timing of the exposure of AEC by siRNA, IFN-β, SPRBD, inhibitors (including SB203580, NSC23766, PH797804, SIS3) and Rx (treatment by desloratadine and monensin) in various combinations.**

[ACE2=angiotensin converting enzyme 2 receptor; ACE2si=ACE2 siRNA; AEC=airway epithelial cells; consi=control siRNA; IFN=interferon; Rx=therapy; SPRBD=spike protein receptor binding domain]

Fig.S3 Impact of NSC23766 and SB203580 on CHST15 and CHST11 expression

**p=0.038**

**p=0.0027**

**p=0.022**

**p=0.02**

**p=8.7x10^-5^**

Exposure to NSC23766, a selective inhibitor of the Rac1-GEF interaction, did not block the SPRBD-induced

increase in expression of CHST15 and CHST11. In contrast, exposure to SB203580, the p38-MAPK inhibitor

significantly reduced their expression.

[IFN=interferon; NSC=NSC23766; SB=SB2-3580’ SPRBD=spike protein receptor binding domain]

Fig.S4 Western blot of phospho(S249)-Rb, total Rb, and tubulin
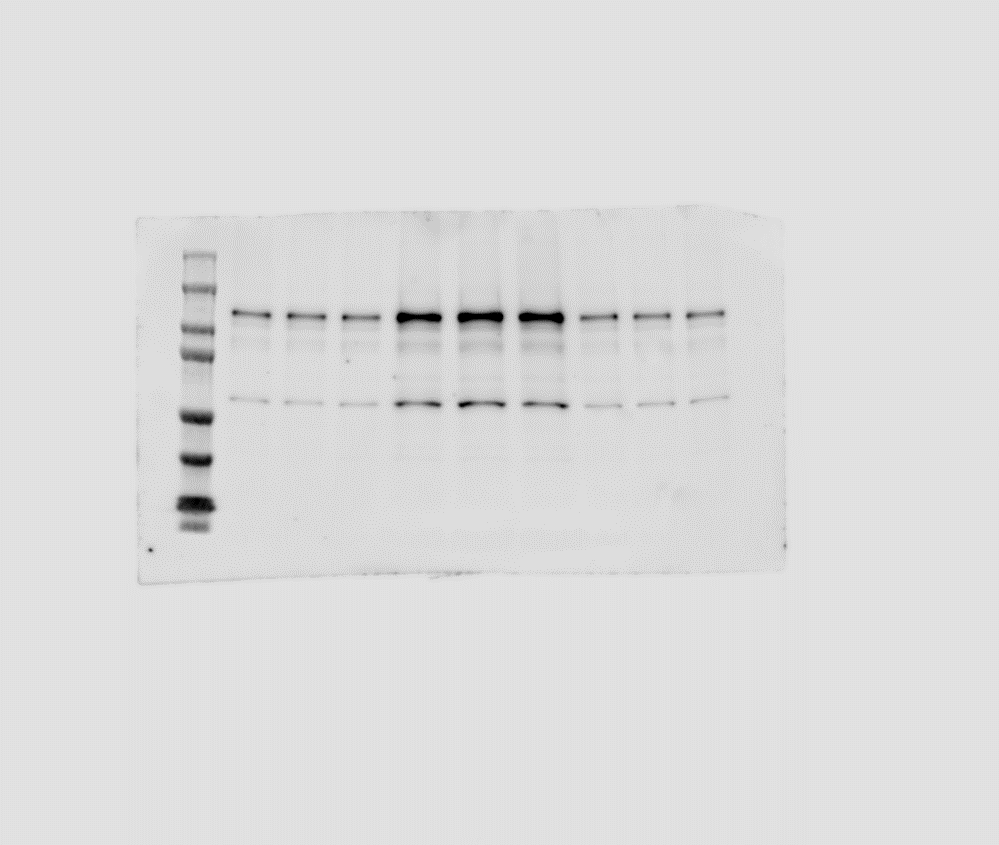

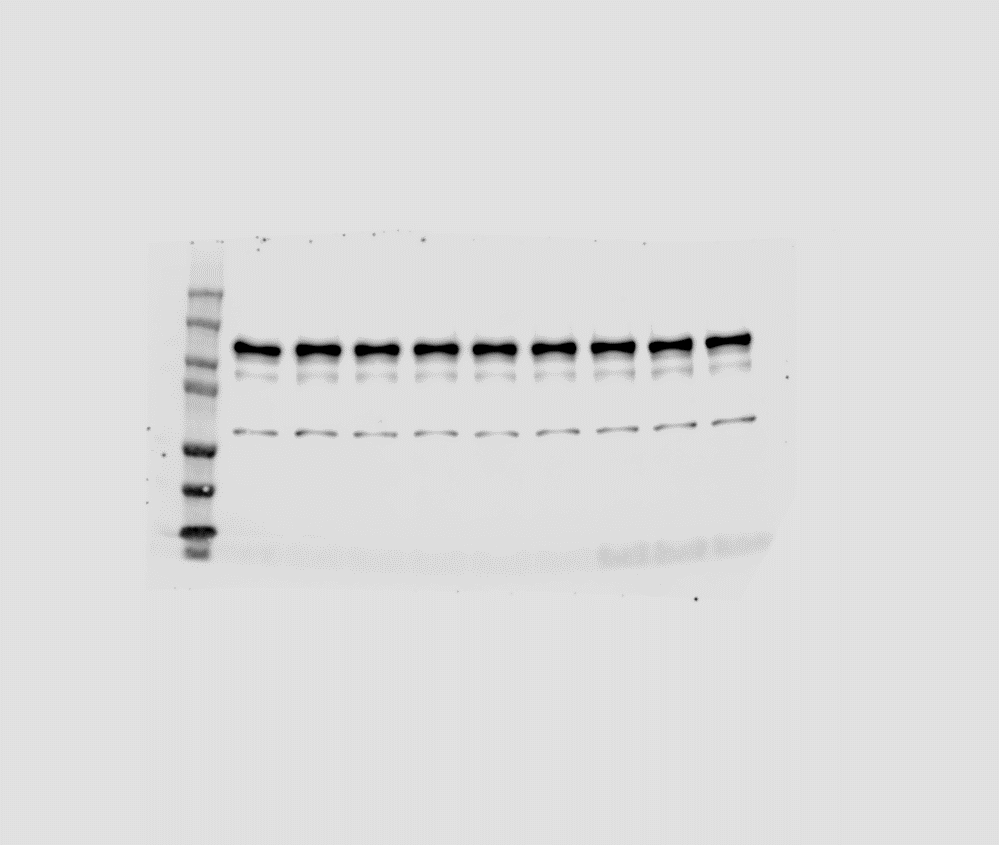


**a**

**Fig.S4 Western blot of phospho(Ser249)-Rb, total Rb, and tubulin**

150kD

100kD

**b**

150kD

100kD


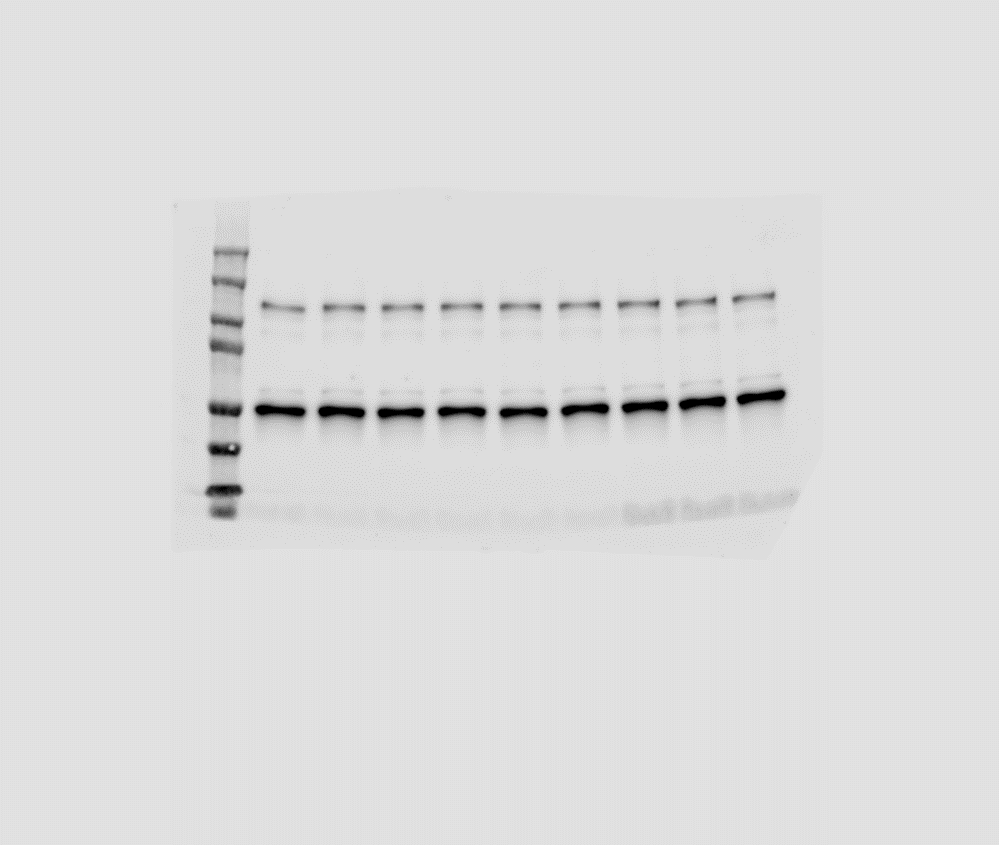


**c**

1. Western blot of phospho(S249)-Rb at ~130 kD.
2. Western blot of total Rb.
3. Western blot of tubulin.
4. Molecular weight key.


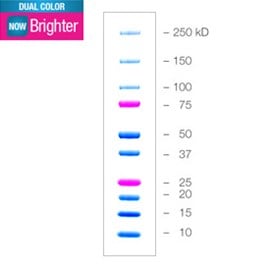


d.

55kD

**Fig.S5 Optical density measurements showing effect of p38MAPK inhibition on E2F1 binding to the ARSB promoter.**

150kD

100kD

**p=0.02**

**p=0.04**

Measurements of optical density indicate that E2F1 binding to the ARSB promoter was markedly reduced following exposure to the SPRBD. This decline was reversed by treatment with SB203580, the p38 MAPK inhibitor.

[IFN=interferon; SB=SB2-3580’ SPRBD=spike protein receptor binding domain]


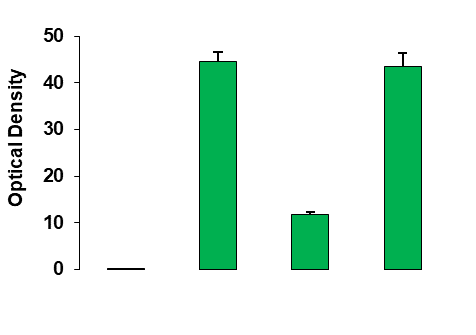


**IgG IFNβ IFNβ+ IFNβ+**

**SPRBD SPRBD+**

**SB**

**Fig.S6 WT Spike Protein Receptor Binding Domain (SPRBD) amino acid sequence**

310 KGIYQTSNFR VQPTESIVRF PNITNLCPFG EVFNATRFAS VYAWNRKRIS NCVADYSVLY NSASFSTFKC YGVSPTKLND LCFTNVYADS FVIRGDEVRQ IAPGQTGKIA DYNYKLPDDF TGCVIAWNSN NLDSKVGGNY NYLYRLFRKS NLKPFERDIS TEIYQAGSTP CNGVEGFNCY FPLQSYGFQP TNGVGYQPYR VVVLSFELLH APATVCGPKK STNLVKNKCV NFNFNGLTGT GVLTESNKKF L 560

**Table S1 ABBREVIATIONS**

ACE2 = angiotensin-converting enzyme 2; converts AngII to Ang(1-7) and AngI into Ang1-9

AEC = airway epithelial cells

Angiotensin(1-7) = Ang1-7= Asp-Arg-Val-Tyr-Ile-His-Pro; binds and activates Mas; is a vasodilator

AngII = angiotensin II = Asp-Arg-Val-Tyr-Ile-His-Pro-Phe; is a vasoconstrictor

ARB = angiotensin receptor blocker

ARSB = arylsulfatase B = N-acetylgalactosamine-4-sulfaltase; removes 4-sulfate group from the non-reducing end of N-acetylgalactosamine 4-sulfate residues of chondroitin 4-sulfate and dermatan sulfate

AT1R = Ang II type 1 receptor

AT2R = Ang II type 2 receptor

C4S = chondroitin 4-sulfate = chondroitin sulfate A

ChIP = chromatin immunoprecipitation

CHST11 = carbohydrate sulfotransferase 11 = chondroitin 4-O-sulfotransferase 1, CHST1

CHST15 = carbohydrate sulfotransferase 15 = N-acetylgalactosamine 4-sulfate 6-O-sulfotransferase, B-cell RAG (Recombination Activating Gene)-associated protein, GALNAc4S-6ST

CHSY1 = chondroitin sulfate synthase 1; has dual glucuronyltransferase and galactosaminyl-transferase activity for chondroitin biosynthesis

consi = control siRNA

COPD = chronic obstructive pulmonary disease

COVID-19 = coronavirus disease 2019

CS = chondroitin sulfate; 4-(D-glucuronate-1,3-D-N-acetylgalactosamine-sulfate)_n_. N-acetylgalactosamine is sulfated at either 4-sulfate, 6-sulfate, 4- and 6- sulfate, or 6-sulfate and glucuronate 2-sulfate

CSE = chondroitin sulfate E = sulfated polysaccharide composed of repeating disaccharides of 4,6-disulfated N-acetylgalactosamine and glucuronic residues linked by alternating β-1,3 and β-1,4 bonds

DS = dermatan sulfate = 4-(D-iduronate-1,3-D-N-acetylgalactosamine-4-sulfate)_n_

E2F1 = transcription factor

ERK = extracellular-regulated kinase

GAG = glycosaminoglycan, includes chondroitin sulfate, dermatan sulfate, heparan sulfate, heparin, hyaluronan, and keratan sulfate

HRP = horseradish peroxidase

IFN-β = interferon-beta

JNK = c-Jun N-terminal kinase

MAPK = mitogen-activated protein kinase

Mas = G-protein-coupled receptor for Ang(1-7), not AngII

MPS VI = mucopolysaccharidosis VI = Maroteaux-Lamy-Syndrome, with congenital deficiency of ARSB

MUS = methylumbelliferyl sulfate; exogenous substrate for measurement of ARSB activity

NSC = NSC23766 = CAS 1177865-17-6; selective inhibitor of Rac1-GEF interaction

p38α = MAPK 14

pRb = phospho-retinoblastoma protein

PAPSS1 = 3'-Phosphoadenosine 5'-Phosphosulfate Synthase 1; sulfate donor for sulfotransferase activity

PAPSS2 = 3'-Phosphoadenosine 5'-Phosphosulfate Synthase 2; sulfate donor for sulfotransferase activity

PH797804 = inhibitor of p38α/β; CAS 1358027-80-1

RAAS = renin-angiotensin-aldosterone system

RAG = Recombination Activating Gene

RAS = renin-angiotensin system

RB = retinoblastoma protein

RBD = receptor-binding domain

sGAG = sulfated glycosaminoglycan, excludes hyaluronan

SARS = Severe Acute Respiratory Syndrome

SB = SB203580 = p38 MAPK inhibitor; CAS No. 152121-47-6; Adezmapimod

SD = standard deviation

si = small interfering siRNA

SIS3 = Smad3 inhibitor, CAS 1009104-85-1; cell permeable selective inhibitor of TGF-β1 dependent Smad3 phosphorylation and Smad3 mediated signaling

SMAD3 = Mothers against decapentaplegic homolog 3 (DPC3); mediator of TGF-β signal transduction

SPRBD = spike protein receptor-binding domain

ST = sulfotransferase

TAB1 = TGF-beta activated kinase (MAP3K7); activates TAK1 and p38alpha

TF = transcription factor

TGF = transforming growth factor
